# Genome Sequence of Cauliflower Mosaic Virus Identified in Earwigs (*Doru luteipes*) through a Metagenomic Approach

**DOI:** 10.1128/genomeA.00043-17

**Published:** 2017-03-16

**Authors:** Márcio Tadeu Godinho, Débora Pires Paula, Arvind Varsani, Simone Graça Ribeiro

**Affiliations:** a Embrapa Recursos Genéticos e Biotecnologia, Brasília, Brazil; b The Structural Biology Research Unit, Department of Clinical Laboratory Sciences, University of Cape Town, Cape Town, South Africa; c The Biodesign Center for Functional Microbiomics, Institute and School of Life sciences, Center for Evolution and Medicine, Arizona State University, Tempe, Arizona, USA

## Abstract

Here we report the first complete genome sequence of a cauliflower mosaic virus from Brazil, obtained from the gut content of the predator earwig (*Doru luteipes*). This virus has a genome of 8,030 nucleotides (nt) and shares 97% genome-wide identity with an isolate from Argentina.

## GENOME ANNOUNCEMENT

Vector-enabled metagenomics (VEM), which allows for the identification of viruses that are vectored by insects and those in higher insect trophic levels, has proved to be a powerful approach for identifying both novel and known plant and animal viruses circulating in various ecosystems (1–7).

As part of a study to identify arthropod trophic interactions, the gut contents from the dermapteran insect predator earwig (*Doru luteipes*) were analyzed as described in reference 8. Ten *D. luteipes* adult individuals were collected in organic farms in Central Brazil, their guts were dissected, and the total DNA was extracted and pooled. This DNA was sequenced on an Illumina MiSeq platform using a TruSeq library (8). The reads were *de novo* assembled using Abyss v1.9 (9) with default parameters and a K-mer = 64. Contigs larger than 800 nucleotides (nt) were analyzed by BLASTx against a local viral database. A contig of 8,030 nt corresponding to the complete sequence of cauliflower mosaic virus (CaMV) was identified with 97% identity with a CaMV (isolate W260 sampled in Argentina; accession number JF809616). Posterior mapping of the Illumina reads using Bowtie (10) to the CaMV *de novo* assembled genome showed a 20× depth. CaMV is a caulimovirus (family: *Caulimoviridae*) and is found worldwide, especially in temperate regions (11). CaMV infects plant species of the Brassicaceae and Solanaceae families, and although it has been reported sporadically in Brazil since the 1960s (compiled by reference 12) this is the first complete genome sequence of a CaMV from Brazil. CaMV is transmitted by *Brevicoryne brassicae*, *Myzus persicae*, and other aphid species in a noncirculative manner.

In the gut of the earwigs, diamondback moth* (Plutella xylostella)* and harlequin ladybird* (Harmonia axyridis)* were identified together with three aphid-specific bacterial symbionts (*Hamiltonella* sp., *Regiella insecticola*, *Serratia symbiotica*) and an *Aphidius* parasitoid (8). Therefore, it is highly likely that CaMV was acquired from an infected plant by aphids or diamondback moth larvae which were preyed on by either the earwigs or by harlequin ladybirds, which appear to have been preyed on by the earwigs.

At higher insect trophic levels coupled with viral metagenomics, predators are very useful for identifying new or known viruses that are present in different species associated within these trophic networks.

### Accession number(s).

The complete genome of CaMV described in this report has been deposited in GenBank under accession number KX434771.
